# Arsenic forms in phytoextraction of this metalloid in organs of 2-year-old *Acer platanoides* seedlings

**DOI:** 10.1007/s11356-018-2739-y

**Published:** 2018-07-20

**Authors:** Sylwia Budzyńska, Zuzanna Magdziak, Piotr Goliński, Przemysław Niedzielski, Mirosław Mleczek

**Affiliations:** 1 Department of Chemistry, Poznań University of Life Sciences, Wojska Polskiego 75, 60-625 Poznań, Poland; 2 Faculty of Chemistry, Adam Mickiewicz University, Umultowska 89B, 61-614 Poznań, Poland

**Keywords:** Dendroremediation, Interactions, Metalloid, Norway maple, Speciation, Uptake

## Abstract

The aim of the study was to estimate the significance of the role of arsenite (As(III)), arsenate (As(V)), and dimethylarsinic acid (DMA) presence in modified Knop medium in the efficiency of phytoextraction of arsenic (As) in *Acer platanoides* root, stem, and leaves. The addition of particular As forms in single, double, and triple experimental systems was associated with a lower increase of seedling biomass compared to control plants (system free of As forms addition). Depending on As forms and their concentration in solution, negative symptoms from slight visible changes (inorganic forms separately or jointly), through smaller and discolored leaves (after DMA addition), and finally to their withering (after high DMA addition) were observed. Changes of color and shape for root systems exposed to particular As forms separately or jointly were also observed, in spite of the fact that there were no significant changes in biomass of seedlings growing in all experimental systems. The highest mean concentrations of As in root, stem, and leaves (590, 70, and 140 mg kg^−1^ dry weight (DW), respectively) were observed in plants growing under different experimental systems. The highest bioconcentration factor values were 10.8 for plants exposed to 0.06 mM of As(III) and DMA, while the highest translocation factor (1.0) was recorded for plants growing under the same As forms (0.6 and 0.06 mM, respectively). The obtained results indicate that the presence of particular As forms not only determines As phytoextraction and transport of this metalloid form but also has a decisive influence on plant morphology and survivability. As regards the practical aspects of phytoremediation, the kind of As forms present in substrate are more important than their total concentration.

**Electronic supplementary material:**

The online version of this article (10.1007/s11356-018-2739-y) contains supplementary material, which is available to authorized users.

## Introduction

Anthropogenic activity is the main cause of the production and disposal of wastes with a potentially high content of toxic heavy metals and metalloids (Sheoran et al. 2015). Arsenic (As) is one of the most dangerous environmental contaminants (Zhang et al. 2017), being a major public health problem which affects hundreds of millions of people worldwide (Carlin et al. 2016). The toxic effect of As depends on many environmental factors but one of the most important is the chemical form of this metalloid (Gamboa-Loira et al. 2017; Makgalaka-Matlala et al. 2008). Inorganic arsenite (As(III)) and arsenate (As(V)) are the dominant forms in water and soil. As(V) is the major form in aerobic conditions, whereas As(III) is the most abundant under a reducing environment (Yanitch et al. 2017). Inorganic As can be converted in plants to two organic forms (monomethylarsonic acid (MMA) and dimethylarsinic acid (DMA)) present in extensive amounts (Mishra et al. 2017; Zhu et al. 2017).

Phytoremediation is one of the most promising ways to decontaminate substrates polluted with heavy metal and/or toxic As (Chaney et al. 1997; Mahar et al. 2016). Especially important is dendroremediation, using trees for their specific morphological traits (Komives and Gullner 2006; Labe and Agera 2017; Schoenmuth and Pestemer 2004). The high survivability and easy adaptation of trees, undisturbed growth, and effective phytoextraction of toxic elements/metalloids make them highly suitable for remediation of substrates polluted by As forms. The chemical structure of As(V) is similar to phosphorus (P); therefore, it can be easily incorporated through phosphate (Pi) transporters into plants (Yanitch et al. 2017). As(III) is similar to sulfur (S), which is uptaken from substrate by nodulin26-like intrinsic aquaporin channels (NIPs) and bounds to the sulfhydryl groups of peptides and proteins inhibiting their activity (Mishra et al. 2017). Due to its high affinity for thiol groups, As(III) may create complexes with glutathione and phytochelatins (PCs) to be stored within the vacuole in order to prevent cell damage (Mishra et al. 2017). It is clear that changes in P and S concentration in plant organs during their exposure to As(V), in the same way as boron (B) and silicon (Si) for As(III), provide crucial information about the possible mechanisms of how these toxic As forms are uptaken (Budzyńska et al. 2017a, b). Uptake of As forms from contaminated substrates to the root system and finally to aboveground plant parts depends not only on mutual interaction between As and other elements but also on the qualitative and quantitative relationships between the major As forms (As(III), As(V), and DMA) available for plants in substrate (Budzyńska et al. 2017b; Kroukamp et al. 2016).

For this reason, the aim of this study was to estimate the influence of the abovementioned As forms on growth (biomass crop), changes in leaf and root morphology, and their transport in *Acer platanoides* organs. This paper is a development of our earlier studies described by Budzyńska et al. (2017b), where the same experiment was performed for *Ulmus laevis* Pall., which allowed us to show the importance of interaction between As forms for the phytoextraction of this metalloid.

## Materials and methods

### Characteristics of seedlings

Experimental materials were 2-year-old seedlings of *A. platanoides* obtained from the Pniewy Forest Division (52° 29′ 04″ N, 16° 15′ 28″ E) in March 2017. The use of *A. platanoides* was a consequence of our previous published and unpublished results, where young seedlings of this tree species with *U. laevis* Pall. and *Betula pendula* Roth. were found to be the most promising for phytoextraction of As or biomass crop (Mleczek et al. 2016). The mean biomass of the plants used in the experiment was 106.8 ± 8.6 g. *A. platanoides* seedlings were grown in cylindrical pots filled with unpolluted soil with pH_1M KCL_ = 7.3. Concentration of potassium (K) and P was 6.23 and 2.62 mg 100 g^−1^ of soil, respectively, while carbon (C) and nitrogen (N) 0.28 and 3.24% of air dry mass, respectively. The seedlings used in the experiment were chosen from a population of some thousand specimens so that the initial material could be as similar as possible. Described differences in their biomass were the effect of the selection of plants with similar root system, stem diameter (1.1–1.3 cm), and height (1.21–1.32 m).

### Experiment design

The greenhouse experiment was undertaken for 3 months. The mean values of concentration of CO_2_, moisture, and temperature were 459.2 ppm, 50.12%, and 22.81 °C, respectively. The minimal and maximal values for these parameters were 293–687 ppm, 25.4–78.5%, and 10.9–38.5 °C, respectively. All data were obtained by data loggers with all parameters automatically recording every hour. Seedlings of *A. platanoides* were cultivated in hydroponic pots (18 × 19 cm, diameter × height) in such a way that 21 experimental systems were set up (Table 1), and each of them was characterized by six plants cultivated in each separate pot (126 plants jointly). All the used seedlings were divided so that the six plants used in each of the 21 experimental systems were similar (according to biomass and height) before their planting into pots. Plants were stabilized using 1.2 kg of ultrapure quartz sand per pot (pH = 7.2, content of SiO_2_ 97%) with a particle size range of 1–3 mm. Modified Knop’s solution (0.8 L per pot) was prepared strictly according to Barabasz et al. (2010) and enriched with the following As forms: As(III), As(V), and DMA in the forms sodium (meta)arsenite (AsNaO_2_), sodium arsenate dibasic heptahydrate (Na_2_HAsO_4_ · 7H_2_O), and dimethylarsinic acid ((CH_3_)_2_As(O)OH). Particular forms of As were added to the final concentrations at 0.06 or 0.6 mM at the start of the experiment (Table 1).

**Table 1:** Characteristics of experiment design

No. of system	System	Addition of As species [mM L^−1^]
1	Control	0
2	As(III)	0.06
3	As(III)	0.6
4	As(V)	0.06
5	As(V)	0.6
6	DMA	0.06
7	DMA	0.6
8	As(III)/As(V)	0.06/0.06
9	As(III)/As(V)	0.6/0.06
10	As(III)/As(V)	0.06/0.6
11	As(III)/DMA	0.06/0.06
12	As(III)/DMA	0.6/0.06
13	As(III)/DMA	0.06/0.6
14	As(V)/DMA	0.06/0.06
15	As(V)/DMA	0.6/0.06
16	As(V)/DMA	0.06/0.6
17	As(III)/As(V)/DMA	0.06/0.06/0.06
18	As(III)/As(V)/DMA	0.6/0.6/0.6
19	As(III)/As(V)/DMA	0.6/0.06/0.06
20	As(III)/As(V)/DMA	0.06/0.6/0.06
21	As(III)/As(V)/DMA	0.06/0.06/0.6

### Sample preparation and analysis

#### Analysis of arsenic

Plant samples were dried at 105 ± 1 °C for 95 h, ground, and extracted using the procedure described in a previous work (Niedzielski et al. 2013). 0.5 g of the sample were put into a test tube containing 5 mL of phosphoric acid (1 mol L^−1^) and extracted in an ultrasonic bath (30 min at ambient temperature). Next, the solution was filtered by paper filter washed in 200 mL of water. The pH of the solution was adjusted at 6–6.5 by the addition of 10 mol L^−1^ solution of NaOH. The total arsenic concentration and concentration of arsenic forms were determined immediately after extraction.

#### Determination of the total arsenic concentration

For determination of the total arsenic concentration, the inductively coupled plasma optical emission spectrometer Agilent 5110 ICP-OES (Agilent, USA) was used. The following conditions were applied: wavelength 188.980 nm, radio frequency (RF) power 1.2 kW, nebulizer gas flow 0.7 L min^−1^, auxiliary gas flow 1.0 L min^−1^, plasma gas flow 12.0 L min^−1^, charge-coupled device (CCD) temperature − 40 °C, accusation time 5 s, and 3 replicates. The detection limit was determined at 0.01 mg kg^−1^ dry weight (DW) as 3-sigma criteria. Accuracy was checked using the standard addition methods due to a lack of reference material for phosphoric acid extraction. Recovery at the level of 80–120% was found as satisfactory.

#### Arsenic speciation studies

**Fig. 1:**
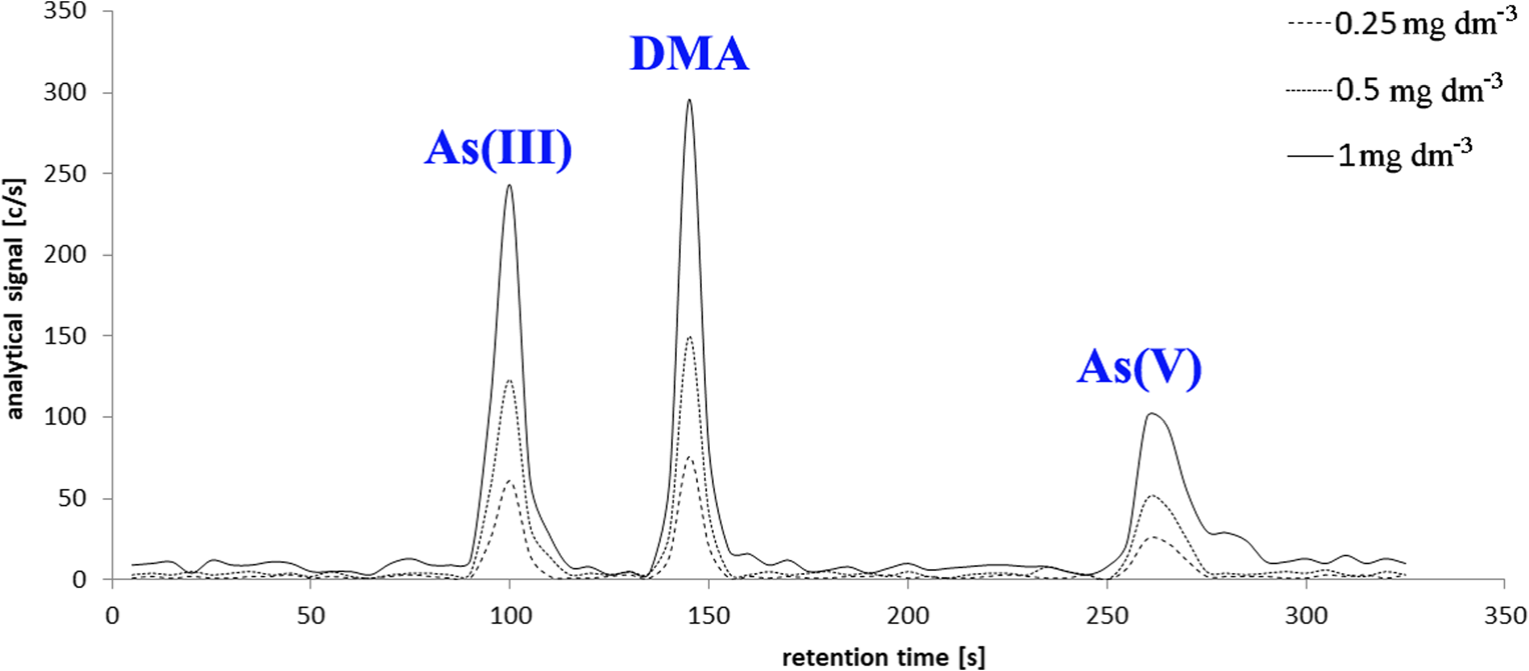
Chromatogram for standards 0.25, 0.5 and 1.0 mg L^−1^ for three arsenic forms: As(III), DMA, and As(V) determined by HPLC-ICP-OES

The arsenic inorganic forms were determined by the hyphenated system of high performance liquid chromatography with inductively coupled plasma optical emission spectrometry detection (HPLC-ICP-OES). An HPLC pump with an anion-exchange column (Supelco, USA) LC-SAX1 (250 mm, 4.6 mm i.d.) was used. The chromatographic run was isocratic at 2.5 mL min^−1^ of phosphate buffer (5 mM Na_2_HPO_4_ and 50 mM KH_2_PO_4_ 2H_2_O) with an injection volume of 200 μL. PEEK (polyetheretherketone) tubing was inserted into a Tygon sleeve for transfer of the eluent from the LC column to the nebulizer of the ICP-OES spectrometer (Agilent 5110 ICP-OES (Agilent, USA)). Three arsenic forms were determined at 188.980 nm with retention time: 100 s for As(III), 148 s for DMA, and 264 s for As(V) (Fig. 1). The content of the remaining organic arsenic forms (As_org_) was calculated from the difference between total arsenic concentration (As_total_) and the sum of inorganic forms of this metalloid and DMA. The determination limits were found at the level of 1.0 mg kg^−1^ for all forms determined. Due to a lack of certified reference materials for arsenic speciation in samples extracted by phosphoric acid, the standard addition method was used for accuracy and traceability studies. Recoveries at the level of 80–120% were found as satisfactory.

#### Determination of B, Ca, K, Mg, Na, P, S, and Si in *A. platanoides* organs

The inductively coupled plasma optical emission spectrometer Agilent 5100 ICP-OES (Agilent, USA) was also applied for B, calcium (Ca), magnesium (Mg), K, sodium (Na), and Si determination. Additionally, phosphorus (P) and sulfur (S) were analyzed according to Cernusak et al. (2010) and Melo et al. (2009). The content of total S was confirmed in selected plant samples with a FLASH 2000 analyzer (Thermo Scientific) with an FPD detector. A synchronous vertical dual view (SVDV) of the plasma was accomplished with dichroic spectral combiner (DSC) technology which allows the axial and radial view to be analyzed simultaneously. The common instrumental conditions were the same as for total As analysis. The wavelengths (nm) were 249.772, 422.673, 766.491, 279.553, 589.592, 253.561, 180.669, and 288.158 for B, Ca, K, Mg, Na, P, S, and Si, respectively.

The detection limits were determined as 3-sigma criteria and were at the level of 0.03, 0.03, 0.03, 0.01, 0.03, 0.03, 0.04, and 0.06 mg kg^−1^ DW for B, Ca, K, Mg, Na, P, S, and Si, respectively. Traceability was checked using the standard addition methods and recoveries at the level 80–120% were found as acceptable. The characteristics of the results obtained for all six elements are presented in Supplementary data (Tables S1–S4).

### Statistical analysis and calculations

All statistical analyses were performed using STATISTICA 12.0 software (StatSoft, USA). To show the differences between As_total_ content in particular organs of *A. platanoides* seedlings growing in the experimental systems, the one-way analysis of variance (ANOVA) followed by the post hoc Tukey HSD test was applied. The same analysis was performed to compare the biomass of seedlings growing in particular experimental systems.

To estimate the real efficiency of *A. platanoides* seedlings growing in Knop medium enriched with particular As forms, the total content of As in whole plant biomass (mg plant^−1^) was calculated as the sum of As content in root, stem, and leaves with consideration of the biomass of these organs. To estimate As phytoextraction efficiency by *A. platanoides* seedlings and their ability to translocate uptaken metalloid to aboveground plant parts, the bioconcentration factor (BCF) and translocation factor (TF) values, respectively, were calculated. The bioconcentration factor was calculated as the ratio of As content in the harvested tree organs to the concentration of this metalloid in substrate, while the TF was calculated as the ratio of As content in aerial plant parts to the content of this metalloid in roots (Ali et al. 2013).

## Results

### Seedling survivability, morphology, and biomass

**Fig. 2:**
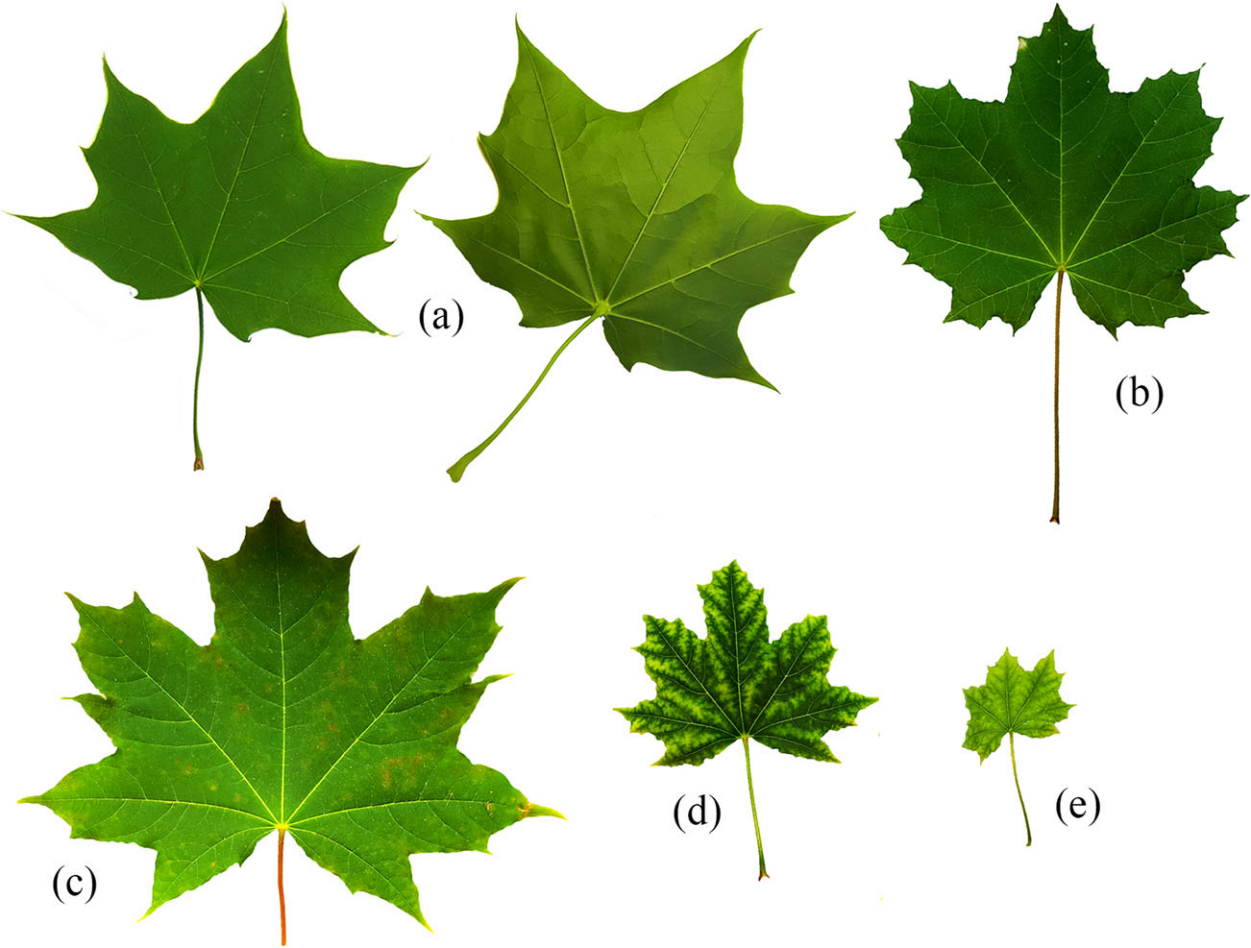
Characteristics of morphology of *A. platanoides* leaves. **a**, **b** Without negative symptoms. **c** With slight visible changes on leaf surface. **d** With clear changes in leaf color. **e** With clear symptoms of As toxicity

Out of all the cultivated *A. platanoides* seedlings in 21 different experimental systems, absence of growth was observed only for plants growing in systems with DMA addition (0.6 mM). In the remaining systems, growth of seedlings was undisturbed, although specific symptoms were observed. Plants growing in the control system (Fig. 2a), like those in systems with the addition of As(III) or As(V), (0.6 or 0.06 mM), were characterized by the full development of leaves (Fig. 2b) without any negative symptoms. The presence of these As forms jointly was generally related with slight visible changes on the leaf surface (Fig. 2c). The addition of DMA (0.06 mM) caused distinct leaf changes; leaves were (Fig. 2d) smaller with discoloration. Leaves of *A. platanoides* seedlings growing in systems with addition of DMA (0.6 mM) after only 9–11 days displayed clear symptoms of As toxicity (Fig. 2e). Leaf falling was observed 17 days after the beginning of the experiment, while clear symptoms of their withering were observed after 34 days of the start point. It is worth underlining that the size of particular leaves was almost identical for plants growing under the same experimental systems.

**Fig. 3:**
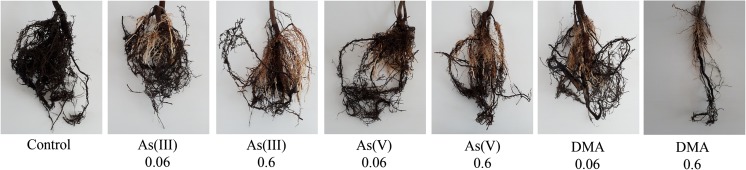
Characteristics of *A. platanoides* roots exposed to the single As forms

**Fig. 4:**
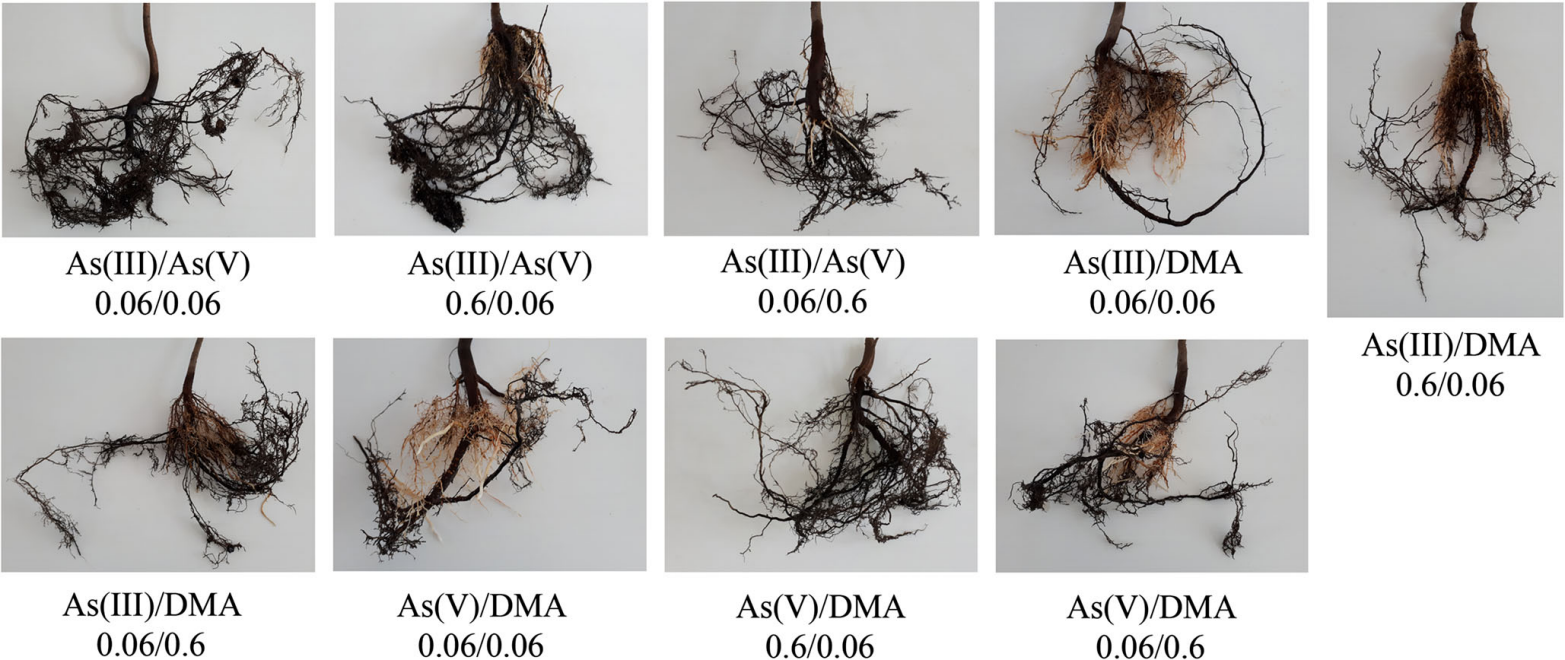
Characteristics of *A. platanoides* roots exposed to two As forms

**Fig. 5:**
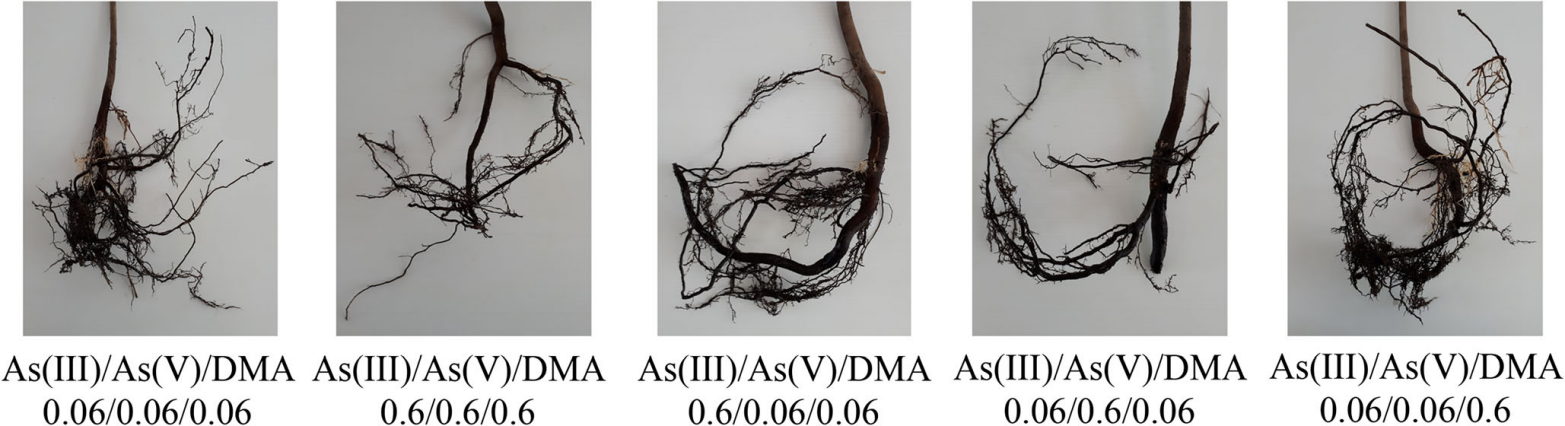
Characteristics of *A. platanoides* roots exposed to three As forms

Changes were also observed for roots of *A. platanoides* seedlings (Figs. 3, 4, 5). Although not all plants can be presented in the figures, and they cannot reflect the tendency of biomass changes presented in Table 2, the purpose of these figures was to show changes in their color and shape in relation to the control. Figures 3, 4, and 5 also show root systems for nonliving plants so as to illustrate the influence of higher concentrations of DMA on plant development.

**Table 2:** Characteristic of plant biomass [g] before and after experiment with percentage of biomass in relation to control seedlings

No. of system	System	Leaves	Stem	Root	Total after	Total before	Increase of biomass	% of control
1	Control	7.2^ab^ ± 1.6	61.1^ab^ ± 2.6	33.4^b^ ± 6.1	101.7^a^ ± 7.9	84.3^a^ ± 4.6	17.4^a^ ± 3.6	–
2	As(III) 0.06	7.2^ab^ ± 4.2	64.8^ab^ ± 8.7	37.6^ab^ ± 4.1	109.6^a^ ± 12.0	97.3^a^ ± 12.9	12.3^abcd^ ± 5.8	57
3	As(III) 0.6	5.1^ab^ ± 1.0	73.4^ab^ ± 6.6	42.0^ab^ ± 15.1	120.5^a^ ± 10.1	107.5^a^ ± 5.7	13.0^abcd^ ± 7.0	27
4	As(V) 0.06	8.1^ab^ ± 3.1	69.7^ab^ ± 11.1	38.4^ab^ ± 7.4	116.2^a^ ± 17.9	105.0^a^ ± 15.6	11.3^abcde^ ± 4.1	51
5	As(V) 0.6	9.4^a^ ± 4.4	77.1^a^ ± 9.5	38.6^ab^ ± 7.3	125.0^a^ ± 13.6	111.3^a^ ± 15.1	13.8^abc^ ± 4.8	33
6	DMA 0.06	11.0^a^ ± 4.3	67.5^ab^ ± 9.4	36.2^ab^ ± 4.9	114.7^a^ ± 12.8	106.4^a^ ± 13.5	8.3^cde^ ± 1.2	63
7	DMA 0.6	npg						
8	As(III)/As(V) 0.06/0.06	9.7^a^ ± 3.1	64.1^ab^ ± 8.2	42.7^ab^ ± 11.9	116.6^a^ ± 14.9	104.9^a^ ± 12.8	11.7^abcde^ ± 2.7	85
9	As(III)/As(V) 0.6/0.06	6.4^ab^ ± 1.4	54.4^b^ ± 11.4	51.5^a^ ± 10.2	112.3^a^ ± 19.4	95.1^a^ ± 17.5	17.1^ab^ ± 3.1	37
10	As(III)/As(V) 0.06/0.6	9.3^a^ ± 3.2	59.8^ab^ ± 14.0	42.6^ab^ ± 5.7	111.7^a^ ± 16.8	100.8^a^ ± 18.4	10.9^abcde^ ± 4.6	40
11	As(III)/DMA 0.06/0.06	9.2^a^ ± 4.8	56.0^b^ ± 14.6	37.2^ab^ ± 10.7	102.4^a^ ± 26.9	91.1^a^ ± 25.8	11.2^abcde^ ± 4.4	38
12	As(III)/DMA 0.6/0.06	9.1^a^ ± 5.0	66.0^ab^ ± 5.5	36.8^ab^ ± 4.5	111.8^a^ ± 8.3	103.6^a^ ± 11.1	8.3^cde^ ± 4.3	22
13	As(III)/DMA 0.06/0.6	npg						
14	As(V)/DMA 0.06/0.06	6.3^ab^ ± 2.9	65.3^ab^ ± 7.9	38.0^ab^ ± 5.2	109.6^a^ ± 3.3	100.4^a^ ± 3.1	9.2^bcde^ ± 3.4	33
15	As(V)/DMA 0.6/0.06	1.5^b^ ± 0.5	67.5^ab^ ± 13.5	29.2^b^ ± 6.1	98.2^a^ ± 13.8	92.6^a^ ± 14.8	5.5^de^ ± 1.5	34
16	As(V)/DMA 0.06/0.6	npg						
17	As(III)/As(V)/DMA 0.06/0.06/0.06	6.8^ab^ ± 2.4	65.3^ab^ ± 11.6	36.9^ab^ ± 16.6	108.9^a^ ± 26.7	98.7^a^ ± 25.4	10.2^abcde^ ± 3.4	39
18	As(III)/As(V)/DMA 0.6/0.6/0.6	npg						
19	As(III)/As(V)/DMA 0.6/0.06/0.06	6.5^ab^ ± 2.1	74.0^ab^ ± 9.0	34.3^ab^ ± 8.1	114.8^a^ ± 15.5	104.8^a^ ± 15.5	9.9^abcde^ ± 2.1	30
20	As(III)/As(V)/DMA 0.06/0.6/0.06	5.9^ab^ ± 4.2	68.0^ab^ ± 10.7	38.4^ab^ ± 3.5	112.2^a^ ± 16.0	108.1^a^ ± 15.2	4.1^e^ ± 1.5	35
21	As(III)/As(V)/DMA 0.06/0.06/0.6	npg						
Mean values for all living specimens		7.4 ± 3.8	65.9 ± 11.1	38.4 ± 9.4	111.6 ± 16.1	100.7 ± 15.8	10.9 ± 5.0	42

Mean values (*n* = 6); identical superscripts denote no significant (*p* < 0.05) difference between mean values in column according to Tukey’s HSD test (ANOVA); *npg* not plant growth

The biomass of seedlings placed in pots with Knop medium enriched with particular As forms before the experiment was similar, with a mean value of 100.7 ± 15.8 g. After the experiment, no significant differences between biomass of *A. platanoides* seedlings growing in particular systems were recorded (Table 2).

The biomass of leaves, stems, and roots of the studied seedlings at the end of the experiment was also similar with some exceptions. The highest mean biomass of leaves was characterized by *A. platanoides* seedlings growing in the DMA (0.06 mM) system (11.0 g), while the lowest mean biomass of seedlings was observed under As(V)/DMA (0.6/0.06 mM), (1.5 g). The mean biomass of leaves for all studied specimens in the experiment was 7.4 g. The biomass of seedling stems growing under As(V) 0.6 mM (77.1 g) was the highest, while the lowest was stated for seedlings growing under the As(III)/As(V), (0.6/0.06 mM) system (54.4 g). In the case of the remaining *A. platanoides* seedlings, the biomass of their stems was similar with a mean value for all living specimens of 65.9 ± 11.1 g. It is worth emphasizing that *A. platanoides* seedlings growing under As(III)/As(V), (0.6/0.06 mM), were simultaneously characterized by the highest mean biomass of their roots (51.5 g). The biomass of the rest of the seedlings was similar, with the lowest value observed for seedlings growing in the As(V)/DMA (0.6/0.06 mM) system—29.2 g. The mean value of root calculated for all studied specimens was 38.4 g.

Control seedlings with the lowest mean biomass at the start of the experiment (84.3 g) were characterized by the highest mean increase of their biomass (17.4 g, which corresponds to 20.6% of the initial biomass). A similar increase was recorded (17.1 g–18%) for seedlings growing in the As(III)/As(V), (0.6/0.06 mM) system. The mean increase of the whole biomass of seedlings calculated for all studied specimens was 10.9 g (11%), which suggests that As presence has an important influence on *A. platanoides* seedling growth and development. This confirmed the lowest biomass increase for seedlings growing under the As(V)/DMA (0.6/0.06 mM) and As(III)/As(V)/DMA (0.06/0.6/0.06 mM) systems (hardly 5.5 and 4.1 g, respectively, which corresponds to 6 and up to 4% of the initial biomass of seedlings).

### Total arsenic content in plant organs

**Fig. 6:**
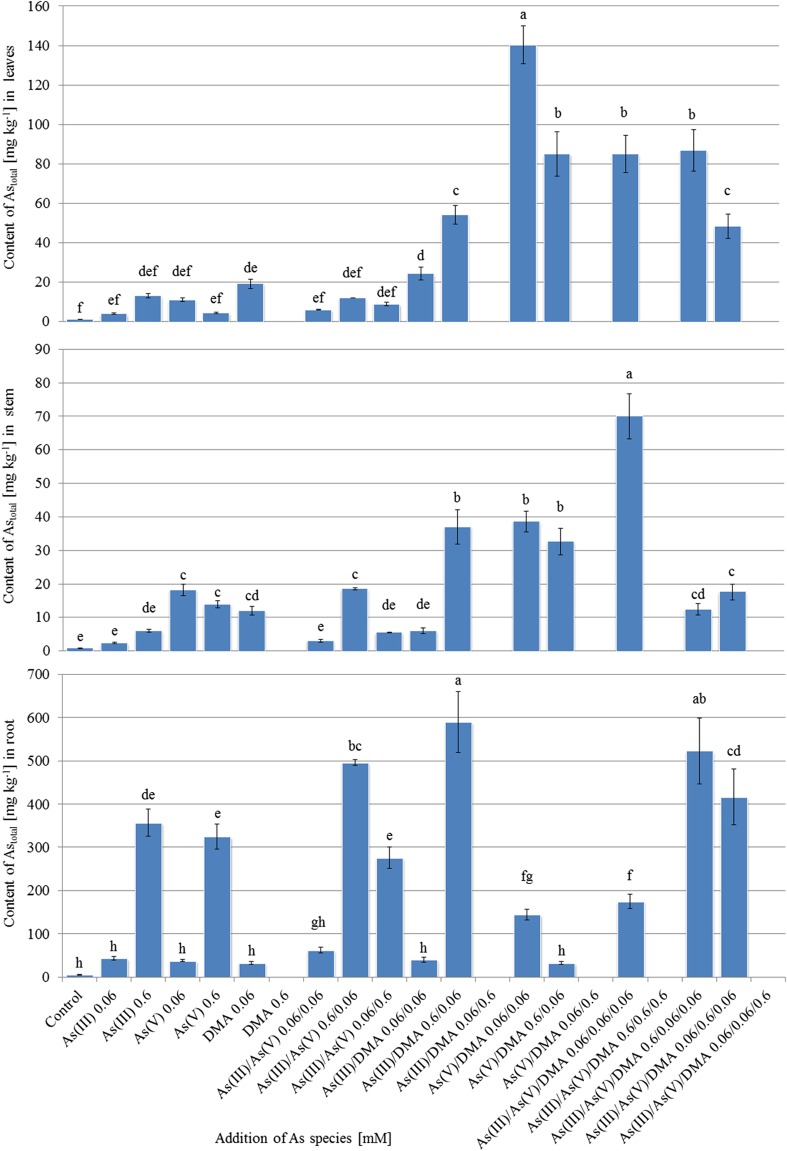
Content [mg kg^−1^ DW] of As_total_ in *A. platanoides* organs

The content of As_total_ in particular organs of *A. platanoides* seedlings was significantly diverse (Fig. 6). As was accumulated mainly in roots, then in leaves, and stem, thus confirming the mean contents of As_total_ calculated for particular organs of all studied seedlings jointly: 222.2, 37.7, and 18.4 mg kg^−1^ DW, respectively. The highest contents of As_total_ were observed in roots of seedlings growing under the As(III)/DMA (0.6/0.06 mM) and As(III)/As(V)/DMA (0.6/0.06/0.06 mM) systems (590 and 523 mg kg^−1^ DW, respectively). Stems of *A. platanoides* seedlings growing under the As(III)/As(V)/DMA (0.06/0.6/0.06 mM) system were characterized by the highest content of As_total_ (70 mg kg^−1^ DW), while leaves of plants exposed to the As(V)/DMA (0.06/0.06 mM) system contained the highest amount of As_total_ (140 mg kg^−1^ DW). The high diversity of As content in organs of seedlings growing in particular experimental systems makes it difficult to estimate the real efficiency of seedlings in As phytoextraction. For this reason, the total content of As in whole biomass was calculated (Table 3).

**Table 3:** Mean content of As_total_ [mg plant^−1^] in *Acer platanoides* L. organs and whole plant biomass

No of system	System	Leaves	Stem	Root	Whole plant
1	Control	0.01 (3.3%)	0.05 (20.8%)	0.17 (75.9%)	0.23
2	As(III) 0.06	0.03 (1.6%)	0.15 (8.5%)	1.62 (89.9%)	1.80
3	As(III) 0.6	0.07 (0.4%)	0.44 (2.8%)	14.98 (96.7%)	15.49
4	As(V) 0.06	0.09 (3.2%)	1.27 (45.2%)	1.45 (51.6%)	2.81
5	As(V) 0.6	0.04 (0.3%)	1.07 (7.8%)	12.54 (91.8%)	11.74
6	DMA 0.06	0.21 (9.6%)	0.81 (36.8%)	1.18 (53.6%)	2.20
7	DMA 0.6	npg			
8	As(III)/As(V) 0.06/0.06	0.06 (2.0%)	0.19 (6.6%)	2.68 (91.4%)	2.93
9	As(III)/As(V) 0.6/0.06	0.08 (0.3%)	1.01 (3.8%)	25.56 (95.9%)	26.64
10	As(III)/As(V) 0.06/0.6	0.08 (0.7%)	0.33 (2.7%)	11.76 (96.6%)	12.17
11	As(III)/DMA 0.06/0.06	0.22 (10.9%)	0.34 (16.4%)	1.49 (72.7%)	2.05
12	As(III)/DMA 0.6/0.06	0.49 (2.0%)	2.44 (9.9%)	21.71 (88.1%)	24.64
13	As(III)/DMA 0.06/0.6	npg			
14	As(V)/DMA 0.06/0.06	0.88 (9.9%)	2.52 (28.4%)	5.49 (61.8%)	8.88
15	As(V)/DMA 0.6/0.06	0.13 (3.9%)	2.20 (67.1%)	0.95 (29.0%)	3.28
16	As(V)/DMA 0.06/0.6	npg			
17	As(III)/As(V)/DMA 0.06/0.06/0.06	0.58 (5.0%)	4.57 (39.4%)	6.45 (55.6%)	11.60
18	As(III)/As(V)/DMA 0.6/0.6/0.6	npg			
19	As(III)/As(V)/DMA 0.6/0.06/0.06	0.56 (2.9%)	0.92 (4.7%)	17.91 (92.4%)	19.39
20	As(III)/As(V)/DMA 0.06/0.6/0.06	0.28 (1.6%)	1.20 (6.8%)	15.98 (91.5%)	17.46
21	As(III)/As(V)/DMA 0.06/0.06/0.6	npg			

Values in round brackets are the percentage participation of As in *A. platanoides* organs in relation to total amount of this metalloid in whole biomass of seedlings; *npg* not plant growth

The highest contents of As_total_ were recorded in seedlings growing under the As(III)/As(V) (0.6/0.06 mM) and As(III)/DMA (0.6/0.06 mM) systems (26.6 and 24.6 g plant^−1^, respectively). The addition of a single As form in higher concentration was associated with a higher content of As in plant biomass. On the other hand, based on systems enriched with two As forms only, phytoextraction of As differed depending on the As form and its concentration in Knop medium (As(III)/As(V) with all three different relationships of concentrations of these forms). The obtained results have shown that content of As was as follows: root>stem>leaves with the exception of seedlings growing in the As(V)/DMA (0.0.6/0.06 mM) system, where As was accumulated mainly in the stem (over 67% of As_total_ in whole plant biomass). Differences between the previously presented efficiency in As phytoextraction in organs is a result of the low biomass of leaves contrary to the stem (Table 2).

### As forms in *A. platanoides* organs

**Fig. 7:**
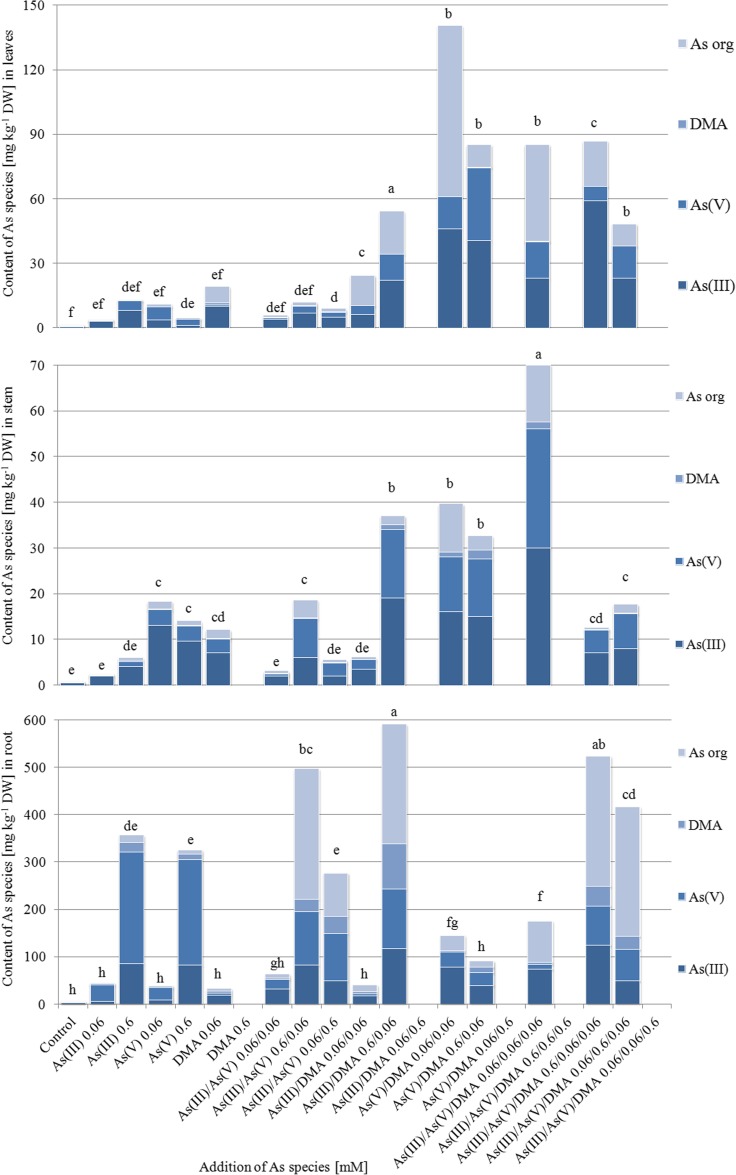
Content [mg kg^−1^ DW] of As(III), As(V), and DMA in *A. platanoides* organs

The addition of As forms to particular experimental systems (Knop mediums) must result in their phytoextraction. The results of As(III), As(V), DMA, and As_org_ content in organs of *A. platanoides* seedlings point to diversity in their phytoextraction in root, stem, and leaves (Fig. 7).

In roots of seedlings exposed to a single As form, As(V) was a dominant form (66–69% of As_total_ in this organ). The addition of two or three As forms jointly was related to the highest content of As_org_ or As(III). The most effective As-absorbing seedlings growing under the As(III)/DMA (0.6/0.06 mM) and As(III)/As(V)/DMA (0.6/0.06/0.06 mM) systems were characterized by the following content of As(III), As(V), DMA, and As_org_ in their roots: 117, 125, 95, and 253 mg kg^−1^ DW, and also 123, 83, 42, and 275 mg kg^−1^ DW, respectively. These values correspond to the following percentage participation of these forms in As_total_ accumulated in roots: 20, 21, 16, and 43%, and 24, 16, 8, and 53%, respectively.

A dominant As form determined in *A. platanoides* stems was As(III), present in all studied systems at a mean content of 55%. Only for stems of seedlings growing under As(III)/As(V) (0.6/0.06 and 0.06/0.6 mM) systems was As(V) a dominant form. The content of particular As forms in the most effective As-absorbing stems of seedlings growing under the As(III)/As(V)/DMA (0.06/0.06/0.06 mM) system was as follows: 30, 26, 1.5, and 12.5 mg kg^−1^ DW, respectively, which corresponds to their percentage participation in As_total_ in this organ: 43, 37, 2, and 18%, respectively. Generally, As(III) was also a dominant form in leaves of *A. platanoides* seedlings. In the case of seedlings growing under the As(V)/DMA (0.06/0.06 mM) system, plants with the highest content of As_total_, the content of As(III), As(V), DMA, and As_org_ was as follows: 46, 15, 0.1, and 79.4 mg kg^−1^ DW, respectively, which corresponds to their percentage participation: 33, 11, 0.07, and 57%, respectively.

Bioconcentration factor values were higher than 1 for all seedlings growing under single As forms added to Knop medium at 0.06 mM, plants growing under all As(III)/DMA and As(V)/DMA systems, and also seedlings under As(III)/As(V)/DMA (0.06/0.06/0.06 and 0.6/0.06/0.06 mM) systems (Table 4). The highest value of BCF (10.79) was recorded for *A. platanoides* seedlings growing under the As(V)/DMA (0.06/0.06 mM) system. It is worth underlining that calculated TF index values were each time < 1, with the exception of plants growing under the As(V)/DMA (0.6/0.06 mM) system, where TF was 1, which coincides with data in Table 3 (over 67% of As_total_ accumulated in stem). The obtained results indicate effective accumulation of As by seedlings growing in selected systems only, with clear limitation in the transport of this metalloid to aerial *A. platanoides* parts.

**Table 4:** Bioconcentration factor (BCF) and translocation factor (TF) values

No. of system	System	BCF	TF
1	Control	–	0.16
2	As(III) 0.06	1.03	0.05
3	As(III) 0.6	0.31	0.02
4	As(V) 0.06	2.93	0.48
5	As(V) 0.6	0.18	0.04
6	DMA 0.06	4.73	0.37
7	DMA 0.6	npg	
8	As(III)/As(V) 0.06/0.06	0.56	0.05
9	As(III)/As(V) 0.6/0.06	0.40	0.04
10	As(III)/As(V) 0.06/0.6	0.14	0.02
11	As(III)/DMA 0.06/0.06	2.37	0.15
12	As(III)/DMA 0.6/0.06	1.25	0.06
13	As(III)/DMA 0.06/0.6	npg	
14	As(V)/DMA 0.06/0.06	10.79	0.27
15	As(V)/DMA 0.6/0.06	1.11	1.00
16	As(V)/DMA 0.06/0.6	npg	
17	As(III)/As(V)/DMA 0.06/0.06/0.06	6.81	0.40
18	As(III)/As(V)/DMA 0.6/0.6/0.6	npg	
19	As(III)/As(V)/DMA 0.6/0.06/0.06	1.20	0.02
20	As(III)/As(V)/DMA 0.06/0.6/0.06	0.58	0.04
21	As(III)/As(V)/DMA 0.06/0.06/0.6	npg	

*npg* not plant growth

## Discussion

The use of trees for effective phytoextraction of As depends mainly on soil chemistry. Bioavailable As forms are uptaken to the root system of plants and this process is generally dependent on the concentration of As forms (Abedin and Meharg 2002; Abedin et al. 2002). On the other hand, effective accumulation of toxic elements or their particular forms is usually related to a decrease of plant biomass or changes in its organ morphology (Srivastawa and Sharma 2014); therefore, it is crucial that the used tree species were characterized by no significant decrease, especially in their aerial organs during the process.

### Arsenic forms and *A. platanoides* biomass and morphology

In our experiment, seedlings of *A. platanoides* were characterized by a similar biomass before and after the experiment but with a smaller increase of treated seedling biomass compared to control plants (Table 2). The presence of As is related to a lower amount of hairy roots, which is more a survival strategy instead of the necessary growth strategy (Bahmani et al. 2016). This situation was observed in our experiment, where exposure of seedlings to As forms resulted in shortening, thinning, and darkening of roots as well as change of shape, size, and color of leaves, depending on the As form present in solution (Abbas et al. 2018; Chaturvedi 2006). Different reactions of plants to As forms were described by Abbas and Meharg (2008), who noted that their presence influences root elongation in different ways and finally affects whole plant growth. This is probably an effect of different mechanisms explained by changes in the concentration of fundamental elements on the uptake of these forms and also their differing speeds of transport from substrate (solution, soil, wastes) to roots (Farooq et al. 2016). The rate of uptake of organic forms from substrate to roots is lower than that of inorganic As(III) or As(V), but after accumulation, organic forms are generally more mobile and transported more quickly to aerial parts (Farooq et al. 2016; Raab et al. 2007). This could explain the higher concentration of As_org_ in leaves than in stem, but not in roots, because in this organ, the highest concentrations of all studied As forms were recorded. Similar relationships were described in our previous paper for *U. laevis* Pall. (Budzyńska et al. 2017b).For both U. *laevis* and *A. platanoides*, higher toxicity was observed for DMA than inorganic forms. Seedlings of both tree species were able to grow in solutions enriched with 0.6 mM of As(III) or As(V) but not in 0.6 mM of DMA. Bissen and Frimmel (2003) found that organic forms of As are less toxic than inorganic, which suggests that higher toxicity of DMA could be related to the faster transport of this form inside the plant by phloem as well as xylem (Ye et al. 2010). There are many contrary data about the higher or lower toxicity of inorganic or organic As forms (Budzyńska et al. 2017b; Carbonell-Barrachina et al. 1998, 1999; Marin 1992) but the fact is that the tree seedlings used in both experiments were unable to survive when growing under DMA (0.6 mM). It is worth underlining that exposure of *A. platanoides* seedlings to DMA was the cause of the lower percentage of the biomass of control plants. Similar observations have been described in literature for many other plants such as *Spartina alterniflora*, *Oryza sativa* L. or *Lycopersicum esculentum* (Burló et al. 1999; Carbonell et al. 1998; Zheng et al. 2012).

Achieving of highest possible biomass of plants used in phytoextraction of toxic elements such as As is important for phytoremediative practice (Hosamane 2012; Yoon et al. 2015). Unfortunately, Masher et al. (2002) have shown that a lower increase of biomass in comparison with control plants is always present below a specific concentration, which suggests that the seedlings studied in our experiment were relatively resistant to the applied concentrations of both organic and inorganic forms. Yoon et al. (2015) pointed out that the presence of DMA was not an inhibitor of plant growth but we suggest that not only are both As forms important but also the concentration of bioavailable forms of this metalloid. Lower or higher toxicity can be also related to the used plants species or many other environmental factors, thanks to that, inorganic As forms may be a less unfavorable influence than DMA on plant biomass (Tlustoš et al. 2002).

The exposure of *U. laevis* L. seedlings to As forms in experimental systems described by Budzyńska et al. (2017b) like those of *A. platanoides* was associated with a different influence of a particular experimental system to increase seedling biomass. Possible differences in response of tree species can be an effect of their growth requirements (rate of nutritional elements uptake), sensitivity to concentration of particular As forms in solution or other factors (Abbas and Meharg 2008). On the other hand, the influence of As forms on seedling biomass was very similar, which suggests that each of them, in the same way as their combinations, has a specific influence on plant growth and development (changes in leaves and root morphology). Changes in the biomass of *A. platanoides* seedlings could also be an effect of changes in the phytoextraction of B and P (Supplementary data), elements important in the uptake of As(III) and As(V), respectively, mainly to plant roots (Budzyńska et al. 2017a; Rasas-Castor et al. 2014; Tu et al. 2004) and for different plants may be present earlier or later from the start point of plant exposure (Singh et al. 2017).

### Phytoextraction of As and this metalloid form

Most plants are able to uptake As mainly in roots (Gomes et al. 2012; Parraga-Aguado et al. 2014), and only selected plant species such as *Pteris vittata* are characterized by effective uptake and translocation of As (Su et al. 2008; Wang et al. 2002). The general model of As uptake in the tree species studied in this and in the previous paper (Budzyńska et al. 2017b) is the same as for the majority of plants described in literature data (Li et al. 2016). The highest concentration of As was observed in roots (BCF > 1 and TF < 1) and accumulation of this metalloid was dependent on its concentration in solution. Increased concentration of particular As forms added to Knop solution individually was associated with their increase in all *A. platanoides* organs. However, addition of two or three As forms jointly caused differences in the efficiency of As_total_ phytoextraction and translocation (Fig. 7). Exposure of plants to As(III) or As(V) not only affects their growth but also As uptake and the translocation of this metalloid form in plants (Makgalaka-Matlala et al. 2008; Raab et al. 2005). Roots usually uptake particular As species in a selective way via specific transporters and distinct pathways (Li et al. 2016); therefore, both the different transport rates of As forms to roots and plant species can explain differences in the accumulation of inorganic and organic forms in plants from the same family and even more so for different tree species (*A. platanoides* and *U. laevis*) (Budzyńska et al. 2017b; Fayiga et al. 2005). The concentration of As(III) in roots of seedlings growing under As(III) or As(V) was almost the same as the concentration of As(V) in plant roots under As(III) or As(V) presence. It is worth underlining that the same relations were present independently of these forms in Knop medium. This suggests that the toxicity of As is related more with the As forms than the concentration of this metalloid (Carbonell-Barrachina et al. 1999; Ye et al. 2010). The presence of selected As forms jointly is also related to higher or lower toxicity of As for plants, which in turn affects root structure and consequently the more or less effective uptake of As forms (Abbas and Meharg 2008). Differences and similarities in phytoextraction of the As forms described in this work and many others are an effect of the use of various plant species and the use of the higher As concentration (Makgalaka-Matlala et al. 2008; Raab et al. 2005). This could be a reason for the limitation of DMA translocation to stems and leaves, as also observed for *U. laevis* (Budzyńska et al. 2017b).

Moreover, the presence of specific transporters and their diversity described in roots and stem of plants can be a factor that may affect the more or less effective transport of particular As forms (Jia et al. 2011). Changes observed for the content of nutritional elements (Ca, K, Mg, Na) but especially B and Si (transporters of As(III)) and also P or Si (Pi) transporters and thiol groups—As(V)) suggest that the probable cause of the observed differences are other pathways (Islam et al. 2015; Li et al. 2016). The decrease of B content in roots and stem, and particularly Si content in all organs of seedlings growing under the majority of experimental systems enriched with As(III) ions in relation to the control, points to the use of B transporters for the transport of As(III) from roots to stem and also Si transporters to transport As(III) from roots to stem and leaves (Ma et al. 2008). A similar situation was observed for P and S for As(V) with the exception of plants growing under As(V) 0.06 mM, which suggests the significant role of other elements present in Knop medium for phytoextraction of As forms by *A. platanoides* seedlings. In our opinion, full knowledge about the real interactions between As forms as well as their changes in relation to nutritional elements is just as important as information about possible transporters and their location in plants (Li et al. 2016; Parmar et al. 2013).

This hydroponic experiment allowed us to show the real response of *A. platanoides* treated with selected As forms. It is worth emphasizing that the obtained results suggest that the specific uptake and translocation of As(III), As(V), or DMA in tree organs is restricted to solution. The response of this tree species growing in soil polluted with As substrates is likely to be different (Goliński et al. 2015; Mleczek et al. 2017) and the obtained result would probably overlap only to a minor degree (Watson et al. 2003). On the other hand, the performance of a hydroponic experiment in a greenhouse, or better in a phytotrone in fully controlled conditions allows the plant response to be determined without the influence of environmental factors (Stolz and Greger 2002).

## Conclusion

Phytoextraction of As and its selected forms depends on numerous environmental factors. Unfortunately, we forget that the same As forms may not be the only influences on the uptake and transport of As but also nutritional and other trace elements will also be involved. Information about the tree response after its exposure to particular As forms (separately and jointly) allowed us to show the transport mechanism of As forms (B or Si transporters for As(III) and Pi or thiol groups for As(V)), but more particularly, the real reaction of plants to specific forms or their concentration. Our results have shown that the presence of higher concentrations of DMA is lethal for 2-year-old tree seedlings. Exposure of these plants to different As forms is strictly related to changes in leaf (smaller, discolored, withered) and root (shortened, black, thinned) morphology. The studied *A. platanoides* are promising subjects for practical application in decontamination of substrate polluted by As, but it is necessary to previously determine the concentration of the As forms studied in this paper which will explain the observed reaction of plants.

## Electronic supplementary material

ESM1 (DOCX 29 kb)
