# Supplementary material for: Arsenic forms in phytoextraction of this metalloid in organs of 2-year-old Acer platanoides seedlings
Source: Environ Sci Pollut Res Int. 2018 Jul 20;25(27):27260–73. doi: 10.1007/s11356-018-2739-y (PMC6132397; doi:10.1007/s11356-018-2739-y)
Supplement: Supplementary file 1 — (DOCX 29 kb) [file 11356_2018_2739_MOESM1_ESM.docx]

Table S1. Content [mg kg^-1^ DW] of B, Ca, K, Na, Mg and Si in *A. platanoides* root growing in particular experimental systems

| No of system | Experimental system | B | Ca | K | Mg | Na | Si |
| --- | --- | --- | --- | --- | --- | --- | --- |
| 1 | Control | 138^a^±8 | 6843^g^±364 | 1161^bcd^±98 | 293^i^±17 | 135^g^±7.2 | 13532^a^±539 |
| 2 | As(III) 0.06 | 114^bc^±10 | 18935^abc^±507 | 1183^bcd^±101 | 435^efgh^±25 | 214^bc^±9.8 | 12382^abc^±575 |
| 3 | As(III) 0.6 | 85^efg^±8 | 14182^f^±398 | 1268^bc^±110 | 515^def^±23 | 229^b^±13.9 | 11995^bc^±804 |
| 4 | As(V) 0.06 | 122^ab^±6 | 17312^de^±421 | 1089^bcde^±76 | 342^hi^±32 | 169^def^±12.5 | 13063^ab^±610 |
| 5 | As(V) 0.6 | 106^bcde^±9 | 18015^cd^±365 | 1314^b^±117 | 447^efg^±13 | 182^cde^±14.8 | 12969^ab^±421 |
| 6 | DMA 0.06 | 91^defg^±8 | 18723^abc^±304 | 1614^a^±146 | 527^de^±29 | 276^a^±7.2 | 10986^cd^±443 |
| 7 | DMA 0.6 | npg | | | | | |
| 8 | As(III)/As(V) 0.06/0.06 | 113^bcd^±5 | 18325^cd^±444 | 978^def^±74 | 418^fgh^±19 | 173^def^±11.1 | 11965^bc^±580 |
| 9 | As(III)/As(V) 0.6/0.06 | 79^fgh^ ±8 | 13968^f^±287 | 865^efg^±63 | 451^efg^±31 | 209^bc^±14.0 | 11709^bcd^±397 |
| 10 | As(III)/As(V) 0.06/0.6 | 96^cdef^±7 | 18412^bcd^±369 | 1292^b^±92 | 385^ghi^±37 | 193^cd^±4.6 | 12169^abc^±527 |
| 11 | As(III)/DMA 0.06/0.06 | 72^ghi^±8 | 17523^de^±449 | 1207^bcd^±123 | 367^ghi^±13 | 152^efg^±13.5 | 11038^cd^±356 |
| 12 | As(III)/DMA 0.6/0.06 | 60^hi^±5 | 16637^e^±332 | 1065^bcde^±75 | 414^gh^±38 | 181^cde^±11.5 | 10894^cd^±421 |
| 13 | As(III)/DMA 0.06/0.6 | npg | | | | | |
| 14 | As(V)/DMA 0.06/0.06 | 113±9^bcd^ | 18859^abc^±285 | 569^hi^±55 | 559^cd^±23 | 143^fg^±12.8 | 11304^cd^±529 |
| 15 | As(V)/DMA 0.6/0.06 | 108±6^bcd^ | 18672^abc^±426 | 407^i^±43 | 638^c^±31 | 68^h^±3.9 | 11258^cd^±348 |
| 16 | As(V)/DMA 0.06/0.6 | npg | | | | | |
| 17 | As(III)/As(V)/DMA 0.06/0.06/0.06 | 69^ghi^±8 | 19486^ab^±269 | 616^ghi^±50 | 789^b^±24 | 86^h^±4.2 | 11008^cd^±278 |
| 18 | As(III)/As(V)/DMA 0.6/0.6/0.6 | npg | | | | | |
| 19 | As(III)/As(V)/DMA 0.6/0.06/0.06 | 53^i^±7 | 18985^abc^±237 | 708^fgh^±83 | 805^b^±42 | 81^h^±10.8 | 10354^d^±452 |
| 20 | As(III)/As(V)/DMA 0.06/0.6/0.06 | 68^ghi^±8 | 19648^a^±285 | 990^cde^±99 | 1197^a^±78 | 164^defg^±13.7 | 11087^cd^±404 |
| 21 | As(III)/As(V)/DMA 0.06/0.06/0.6 | npg | | | | | |

Mean values (n = 6); identical superscripts (a, b, c…) denote no significant (p<0.05) difference between mean values in column according to Tukey’s HSD test (ANOVA); npg – not plant growth

Table S2. Content [mg kg^-1^ DW] of B, Ca, K, Na, Mg and Si in *A. platanoides* stem growing in particular experimental systems

| No of system | Experimental system | B | | Ca | | K | | Mg | | Na | | Si |
| --- | --- | --- | --- | --- | --- | --- | --- | --- | --- | --- | --- | --- |
| 1 | Control | 47.9^a^±1.1 | 13762^ef^±541 | | 615^h^±35 | | 1551^k^±72 | | 985^fg^±34 | | 625^a^±21 | |
| 2 | As(III) 0.06 | 42.2^ab^±2.2 | 16788^b^±617 | | 1874^bcde^±129 | | 2245^i^±86 | | 957^fg^±40 | | 473^bcde^±30 | |
| 3 | As(III) 0.6 | 33.6^cd^±1.7 | 16149^bc^±374 | | 2007^bc^±167 | | 2801^fg^±207 | | 370^h^±40 | | 418^defg^±28 | |
| 4 | As(V) 0.06 | 45.9^a^±1.9 | 11472^g^±353 | | 2142^ab^±140 | | 1916^j^±58 | | 247^h^±21 | | 546^ab^±36 | |
| 5 | As(V) 0.6 | 43.6^ab^±1.7 | 13639^ef^±415 | | 1704^cdef^±132 | | 3095^def^±83 | | 1783^b^±69 | | 518^bc^±22 | |
| 6 | DMA 0.06 | 37.9^bc^±1.2 | 17965^a^±278 | | 2418^a^±160 | | 2368^hi^±66 | | 1123^ef^±79 | | 445^cdef^±33 | |
| 7 | DMA 0.6 | npg | | | | | | | | | | |
| 8 | As(III)/As(V) 0.06/0.06 | 42.9^ab^±2.3 | 13753^ef^±227 | | 1688^cdef^±129 | | 2571^fg^±74 | | 1077^fg^±58 | | 478^bcde^±38 | |
| 9 | As(III)/As(V) 0.6/0.06 | 33.6^cd^±2.9 | 13249^f^±490 | | 1463^f^±147 | | 2927^ef^±108 | | 1614^bc^±73 | | 436^cdef^±24 | |
| 10 | As(III)/As(V) 0.06/0.6 | 44.1 ^ab^±2.3 | 15295^cd^±359 | | 1359^fg^±109 | | 3627^c^±101 | | 2279^a^±95 | | 497^bcd^±30 | |
| 11 | As(III)/DMA 0.06/0.06 | 30.7^cde^±3.3 | 13452^f^±379 | | 1684^cdef^±156 | | 2088^ij^±42 | | 878^g^±61 | | 403^efg^±17 | |
| 12 | As(III)/DMA 0.6/0.06 | 27.8^de^±3.0 | 14764^de^±217 | | 1425^f^±76 | | 3362^cd^±102 | | 2104^a^±99 | | 362^fg^±31 | |
| 13 | As(III)/DMA 0.06/0.6 | npg | | | | | | | | | | |
| 14 | As(V)/DMA 0.06/0.06 | 42.5^ab^±2.8 | 7995^i^±414 | | 1486^ef^±155 | | 5306^a^±77 | | 1425^cd^±97 | | 475^bcde^±23 | |
| 15 | As(V)/DMA 0.6/0.06 | 43.1^ab^±2.6 | 10409^gh^±385 | | 1915^bcd^±171 | | 3198^de^±83 | | 1294^de^±52 | | 496^bcd^±13 | |
| 16 | As(V)/DMA 0.06/0.6 | npg | | | | | | | | | | |
| 17 | As(III)/As(V)/DMA 0.06/0.06/0.06 | 30.7^cde^±3.3 | 10968^gh^±344 | | 1694^cdef^±132 | | 3510^c^±110 | | 1085^efg^±70 | | 416^defg^±39 | |
| 18 | As(III)/As(V)/DMA 0.6/0.6/0.6 | npg | | | | | | | | | | |
| 19 | As(III)/As(V)/DMA 0.6/0.06/0.06 | 24.5^e^±2.6 | 7542^i^±217 | | 995^gh^±125 | | 3619^c^±94 | | 1785^b^±84 | | 338^fg^±21 | |
| 20 | As(III)/As(V)/DMA 0.06/0.6/0.06 | 31.8^cd^±2.2 | 10056^h^±339 | | 1548^def^±80 | | 4027^b^±103 | | 1740^b^±101 | | 408^efg^±25 | |
| 21 | As(III)/As(V)/DMA 0.06/0.06/0.6 | npg | | | | | | | | | | |

Mean values (n = 6); identical superscripts (a, b, c…) denote no significant (p<0.05) difference between mean values in column according to Tukey’s HSD test (ANOVA); npg – not plant growth

Table S3. Content [mg kg^-1^ DW] of B, Ca, K, Na, Mg and Si in *A. platanoides* leaves growing in particular experimental systems

| No of system | Experimental system | B | Ca | K | Mg | Na | Si |
| --- | --- | --- | --- | --- | --- | --- | --- |
| 1 | Control | 37.4^a^±1.6 | 13804^a^±193 | 18904^cde^±892 | 5013^efg^±218 | 678^h^±15 | 158^a^±12 |
| 2 | As(III) 0.06 | 35.1^abc^±1.8 | 9314^b^±577 | 11395^ij^±1082 | 5319^cdef^±223 | 1296^bc^±59 | 119^bcd^±9 |
| 3 | As(III) 0.6 | 31.9^abcd^±1.9 | 6809^def^±484 | 15216^gh^±1234 | 5425^bcdef^±94 | 1480^a^±64 | 107^d^±11 |
| 4 | As(V) 0.06 | 36.5^ab^±1.2 | 5925^def^±491 | 9233^j^±315 | 5226^defg^±62 | 1043^def^±72 | 143^abc^±17 |
| 5 | As(V) 0.6 | 35.2^abc^±2.1 | 6607^def^±529 | 11705^i^±891 | 4925^efg^±113 | 1325^abc^±48 | 147^ab^±8 |
| 6 | DMA 0.06 | 33.7^abc^±2.0 | 8175^bc^±471 | 16221^fg^±684 | 5188^defg^±130 | 1344^ab^±68 | 110^d^±10 |
| 7 | DMA 0.6 | npg | | | | | |
| 8 | As(III)/As(V) 0.06/0.06 | 32.8^abcd^±1.8 | 7141^cd^±418 | 13716^hi^±960 | 4582^gh^±153 | 1410^ab^±52 | 115^cd^±12 |
| 9 | As(III)/As(V) 0.6/0.06 | 30.7^cd^±1.6 | 5537^f^±303 | 16189^fg^±528 | 4232^hi^±137 | 1108^de^±57 | 109^d^±4 |
| 10 | As(III)/As(V) 0.06/0.6 | 32.4^abcd^±1.8 | 7182^cd^±495 | 15739^gh^±1282 | 3880^i^±116 | 1072^def^±63 | 117^cd^±7 |
| 11 | As(III)/DMA 0.06/0.06 | 31.1^bcd^±1.6 | 6063^def^±339 | 23046^ab^±339 | 4819^fgh^±99 | 1099^de^±66 | 111^d^±5 |
| 12 | As(III)/DMA 0.6/0.06 | 30.5^cd^±1.7 | 5712^ef^±389 | 18486^def^±853 | 5947^abc^±200 | 1165^cd^±87 | 105^d^±12 |
| 13 | As(III)/DMA 0.06/0.6 | npg | | | | | |
| 14 | As(V)/DMA 0.06/0.06 | 33.5^abc^±1.4 | 7039^cd^ ±451 | 20239^cd^±755 | 5307^cdef^±130 | 924^fg^±52 | 120^bcd^±14 |
| 15 | As(V)/DMA 0.6/0.06 | 32.8^abcd^±1.4 | 7105^cd^ ±379 | 24478^a^±527 | 6054^ab^±122 | 863^g^±31 | 118^bcd^±4 |
| 16 | As(V)/DMA 0.06/0.6 | npg | | | | | |
| 17 | As(III)/As(V)/DMA 0.06/0.06/0.06 | 32.1^abcd^±2.5 | 6689^def^±482 | 21045^bc^±427 | 6236^a^±44 | 967^efg^±42 | 112^d^±6 |
| 18 | As(III)/As(V)/DMA 0.6/0.6/0.6 | npg | | | | | |
| 19 | As(III)/As(V)/DMA 0.6/0.06/0.06 | 27.5^d^±2.1 | 6561^def^±274 | 17290^efg^±420 | 5548^bcde^±657 | 859^g^±30 | 101^d^±4 |
| 20 | As(III)/As(V)/DMA 0.06/0.6/0.06 | 32.7^abcd^±3.1 | 6950^cde^±353 | 16475^fg^±471 | 5683^abcd^±130 | 901^fg^±67 | 109^d^±7 |
| 21 | As(III)/As(V)/DMA 0.06/0.06/0.6 | npg | | | | | |

Mean values (n = 6); identical superscripts (a, b, c…) denote no significant (p<0.05) difference between mean values in column according to Tukey’s HSD test (ANOVA); npg – not plant growth

Table S4. Content of P [g kg^-1^ DW] and S [%] in root and stem of *A. platanoides* growing in particular experimental systems

| No of system | Experimental system | Root | | Stem | |
| --- | --- | --- | --- | --- | --- |
|  |  | P | S | P | S |
| 1 | Control | 0.85^a^±0.04 | 0.72^a^±0.03 | 0.93^a^±0.09 | 0.62^a^±0.03 |
| 2 | As(III) 0.06 | 0.80^ab^±0.05 | 0.66^abc^±0.02 | 0.89^ab^±0.02 | 0.58^ab^±0.02 |
| 3 | As(III) 0.6 | 0.76^abc^±0.04 | 0.64^abc^±0.02 | 0.87^ab^±0.02 | 0.56^abc^±0.02 |
| 4 | As(V) 0.06 | 0.70^bcd^±0.05 | 0.57^cdefg^±0.03 | 0.84^abc^±0.04 | 0.54^abcde^±0.03 |
| 5 | As(V) 0.6 | 0.61^defg^±0.03 | 0.52^efgh^±0.03 | 0.76^cdefg^±0.03 | 0.50^bcdef^±0.04 |
| 6 | DMA 0.06 | 0.77^abc^±0.03 | 0.67^ab^±0.02 | 0.89^ab^±0.04 | 0.55^abcd^±0.03 |
| 7 | DMA 0.6 | npg | | | |
| 8 | As(III)/As(V) 0.06/0.06 | 0.68^bcde^±0.05 | 0.57^cdefg^±0.04 | 0.84^abc^±0.03 | 0.49^bcdefg^±0.04 |
| 9 | As(III)/As(V) 0.6/0.06 | 0.67^cdef^±0.03 | 0.59^bcdef^±0.02 | 0.82^bcd^±0.04 | 0.47^cdefgh^±0.04 |
| 10 | As(III)/As(V) 0.06/0.6 | 0.58^defg^±0.04 | 0.53^efgh^±0.03 | 0.71^efg^±0.06 | 0.43^fgh^±0.02 |
| 11 | As(III)/DMA 0.06/0.06 | 0.65^cdef^±0.03 | 0.61^bcde^±0.02 | 0.81^bcde^±0.03 | 0.55^abcd^±0.02 |
| 12 | As(III)/DMA 0.6/0.06 | 0.63^defg^±0.05 | 0.63^abcd^±0.05 | 0.79^bcdef^±0.05 | 0.53^abcde^±0.02 |
| 13 | As(III)/DMA 0.06/0.6 | npg | | | |
| 14 | As(V)/DMA 0.06/0.06 | 0.60^defg^±0.07 | 0.54^defgh^±0.01 | 0.74^cdefg^±0.04 | 0.45^efgh^±0.03 |
| 15 | As(V)/DMA 0.6/0.06 | 0.52^gh^±0.03 | 0.48^gh^±0.04 | 0.66^g^±0.03 | 0.40^gh^±0.05 |
| 16 | As(V)/DMA 0.06/0.6 | npg | | | |
| 17 | As(III)/As(V)/DMA 0.06/0.06/0.06 | 0.56^efgh^±0.04 | 0.50^fgh^±0.04 | 0.72^defg^±0.01 | 0.46^defgh^±0.05 |
| 18 | As(III)/As(V)/DMA 0.6/0.6/0.6 | npg | | | |
| 19 | As(III)/As(V)/DMA 0.6/0.06/0.06 | 0.55^fgh^±0.02 | 0.51^fgh^±0.04 | 0.69^fg^±0.04 | 0.45^efgh^±0.03 |
| 20 | As(III)/As(V)/DMA 0.06/0.6/0.06 | 0.45^h^±0.05 | 0.45^h^±0.03 | 0.54^h^±0.04 | 0.39^h^±0.03 |
| 21 | As(III)/As(V)/DMA 0.06/0.06/0.6 | npg | | | |

Mean values (n = 6); identical superscripts (a, b, c…) denote no significant (p<0.05) difference between mean values in column according to Tukey’s HSD test (ANOVA); npg – not plant growth
